# Supplementary material for: Hepatitis B vaccine uptake and associated factors among pregnant women attending antenatal care at Gulu Regional Referral Hospital, Northern Uganda
Source: PLoS One. 2026 Jun 2;21(6):e0350568. doi: 10.1371/journal.pone.0350568 (PMC13229301; doi:10.1371/journal.pone.0350568)
Supplement: Attachment — Submitted filename: Response to Reviewers.docx [file pone.0350568.s003.docx]

Makerere University,

School of Public Health

11.04.2026

**The Academic Editor**

**PLOS ONE**

Dear Editors,

We appreciate the opportunity to have our manuscript reviewed and for the valuable comments provided. We have fully addressed the comments as indicated below.

| **Comment** | **Response** | **Page No.** |
| --- | --- | --- |
| **Reviewer one** | | |
| 1. Throughout the manuscript, avoid the use of "Hep B" and abbreviate "Hepatitis B virus " as "HBV." | Thank you for this comment. The whole document has been reviewed once again and replaced Hep B with HBV as you guided. | All document |
| 2. Update statistics to 2026 or 2025; the numbers have changed | Thank you for this comment, and this is well considered throughout the document. | All document |
| 3. Include REC/UNCST approval numbers on the ethics sections. | Thank you for this comment. The study protocol number is **MakSPH-REC 379**, and I have included it in the ethics section as you recommended. | 29 |
| 4. What was the mean age of the participants? | Thank you for this comment. The mean age of the participants was 26years, with the standard deviation of 5.9 years. I have included this in the table. | 12 |
| 5. There is an additional study on HBV in Uganda not cited "Gwokyalya AM, Nakityo I, Twinamasiko N, Kihumuro RB, Isiko I, Kasoma RM, Bongomin F. Hepatitis B Vaccination History among medical intern doctors and nurses in three national referral hospitals in Uganda. Afr Health Sci. 2025 Dec;25(4):68-74. doi: 10.4314/ahs.v25i4.8. PMID: 41669128; PMCID: PMC12883965." | Thank you, dear reviewer, for this suggestion. We have read through this paper and considered it under a section in methodology. | Page 8 |
| 6. Ensure the literature review to inform the introduction and discussion sections is up-to-date. | Thank you for this comment, and this is well considered in all the documents. | All document |
| **Reviewer 2** | | |
| Definition of Vaccine Uptake The manuscript defines uptake as “receipt of at least one dose,” yet the abstract and conclusion emphasize “complete vaccination” (three doses). Please ensure consistency throughout the manuscript and clearly distinguish between “any-dose uptake” and “complete series coverage.” | Thank you for this important observation. We have revised the manuscript to ensure consistency in the definition and reporting of HBV vaccine uptake. Specifically, we have clearly distinguished between “any-dose uptake” (receipt of at least one dose) and “complete vaccination” (receipt of all three doses) throughout the manuscript.  In the abstract, the results and conclusion have been updated to consistently reflect “any-dose uptake” in line with our operational definition. References to complete vaccination have been removed from the abstract to avoid ambiguity.  **Before corrections were made, the results and conclusion of the abstract read as,**  ***Results:*** *More than half (53%:229/430) of the pregnant mothers had received at least a dose of the HBV vaccine. Of the vaccinated participants, 20.9% (90/229) had received all three doses of the three-dose HBV vaccine. Factors statistically significantly associated with HBV vaccine uptake included age (APR=1.10, 95% CI: 1.02-1.18), knowledge about HBV (APR=1.06, 95% CI: 1.01-1.10), and healthcare worker communication (APR=1.17, 95% CI: 1.04-1.32).*  ***Conclusion:*** *Only 1 in 5 pregnant mothers had complete HBV vaccination. Age, knowledge, and healthcare communication influenced vaccine uptake. There is a need to provide adequate health education regarding the importance of completing the HBV vaccine to pregnant women and community members to improve vaccine outcomes.*  **After correction, the results and conclusion of the abstract now read,**  ***Results:*** *More than half (53%:229/430) of the pregnant mothers had received at least a dose of the HBV vaccine. Factors statistically significantly associated with HBV vaccine uptake included age (APR=1.10, 95% CI: 1.02-1.18), knowledge about HBV (APR=1.06, 95% CI: 1.01-1.10), and healthcare worker communication (APR=1.17, 95% CI: 1.04-1.32).*  ***Conclusion:*** *More than half of pregnant mothers had received at least a dose of the HBV vaccination. Age, knowledge, and healthcare communication influenced vaccine uptake. There is a need to provide adequate health education regarding the importance of completing the HBV vaccine to pregnant women and community members to improve vaccine outcomes.*  Under study variables and measurement, we have updated it to consistently reflect “any-dose uptake” in line with our operational definition. References to complete vaccination have been removed to avoid ambiguity. This is now how it reads concerning vaccine uptake,  *“The primary study outcome variable was HBV vaccine uptake, which was recorded as a binary variable (Yes = 1, No = 0) based on participants' responses. Vaccination status for pregnant mothers who had received any of the HBV doses, either before or during pregnancy, was verified through vaccination cards or doctors’ records. Those with no vaccination were classified as not vaccinated following the criteria used in similar studies (Eleje, Akaba et al. 2021).”* | Page 2, 8 and 27. |
| **Sampling Strategy** |  |  |
| The Methods section describes systematic sampling from ANC attendance lists with replacement of refusals, although the study is described as “randomly selected.” Please clarify the exact sampling approach and revise terminology for accuracy. | Thank you for this important clarification. We have revised the manuscript to accurately reflect the sampling approach used in the study. Specifically, the term “randomly selected” has been replaced with “systematic sampling” to align with the actual procedure applied during participant selection from the ANC attendance lists, including the replacement of refusals.  **Before it was reading,**  ***Methods:*** *This was a cross-sectional study. Data were collected from 430 participants who were randomly selected. Data were collected using an electronic questionnaire built on the Kobo Collect server and analyzed using STATA version 16.0. Prevalence ratios (PR) were calculated using a modified Poisson regression analysis to determine the association between HBV vaccine uptake and the predictors.*  **Now it reads,**  ***Methods:*** *This was a cross-sectional study. Data were collected from 430 participants who were selected by systematic sampling. Data were collected using an electronic questionnaire built on the Kobo Collect server and analyzed using STATA version 16.0. Prevalence ratios (PR) were calculated using a modified Poisson regression analysis to determine the association between HBV vaccine uptake and the predictors.* | Page 2 |
| **Study Population and Generalizability** |  |  |
| It should be explicitly stated that the sample represents pregnant women attending ANC at Gulu Regional Referral Hospital (GRRH), not all pregnant women in Northern Uganda. This limitation should be clearly acknowledged in the Discussion. | Thanks for this comment.  **Before corrections were made**  *The study included all pregnant women attending ANC at GRRH during the study period.*  **After corrections were made**  *The study included pregnant women attending ANC at GRRH who were sampled during the study period.*  We have highlighted it in the limitation and strength of the study  *The study's focus on pregnant women attending ANC at GRRH limits its generalizability to the wider region or country. Nonetheless, the findings provide valuable insights specific to this population and region and can serve as a basis for future, more extensive studies.* | Page 7 and 27 |
| **Knowledge Assessment and Scoring** |  |  |
| Knowledge was assessed using 10 items (including multi-response questions), generating a 0–16 score, which was dichotomized at the median into “good” and “poor.” Please clarify: How multi-response items were scored (e.g., one point per correct option regardless of incorrect selections, or whether incorrect responses were penalized).  The rationale for dichotomizing at the median | Thank you, dear reviewer, for this insight. First, regarding the scoring of multi-response items: For questions that allowed multiple correct responses, each correct option selected by the respondent was awarded one (1) point, while incorrect options selected were assigned zero (0) points. No negative marking or penalty was applied for incorrect selections. Therefore, respondents could accumulate more than one point per item depending on the number of correct responses identified. This approach is consistent with similar knowledge assessment methodologies used in public health research and allows for a more nuanced capture of respondents’ knowledge levels across multiple domains  Second, regarding the rationale for dichotomizing the knowledge score at the median:  The decision to dichotomize knowledge into “good” and “poor” categories using the median score was based on both methodological and practical considerations. The median provides a data-driven, distribution-sensitive cutoff that is appropriate in the absence of universally established or validated thresholds for HBV knowledge in similar populations. This approach has been applied in comparable studies (DeCoster et al., 2011; Tatsilong et al., 2016), allowing for consistency with existing literature. Additionally, dichotomization facilitates interpretability and ease of analysis, particularly for logistic regression models where a binary outcome improves clarity in estimating associations with HBV vaccine uptake. | Page 10 |
| Consider conducting a sensitivity analysis by modeling knowledge as a continuous variable or categorizing it into tertiles. At a minimum, acknowledge the potential loss of information from dichotomization as a limitation. | Thanks for this comment, dear reviewer. Upon conducting a sensitivity analysis by modelling knowledge as a continuous variable, knowledge remained significantly associated with the outcome (HBV vaccine uptake), with a coefficient of 1.01 (1.00-1.01), P = 0.010. |  |
| Regression Model Clarity and Variable Selection The use of modified Poisson regression with robust variance to estimate prevalence ratios is appropriate. However:  The Methods section inconsistently states that variables with p<0.2 (and elsewhere p<0.05) were included in the multivariable model. Please standardize this criterion | Thank you for this comment. We have clearly corrected to p<0.2 for consistency. It now reads,  *All variables whose p-values were less than 0.2 were included in the final model. Therefore, the narrative for Table 5 only reports variables that were associated with the outcome. Adjusted prevalence ratios at a 95% confidence interval were used to measure the strength of the association, and variables whose p-values were less than 0.05 were statistically significant. Variables that were found to be statistically significant (p-value < 0.2) in the bivariate analysis were included in the initial model.* | Page 10 |
| Table 5 indicates adjustment only for age, religion, education, and knowledge. Clarify whether other covariates were considered and the final model-building strategy. | In the section on data management and analysis, under methodology, we noted that all variables whose p-values were less than 0.2 were included in the final model. Therefore, the narrative for Table 5 only reports variables that were associated with the outcome. Adjusted prevalence ratios at a 95% confidence interval were used to measure the strength of the association, and variables whose p-values were less than 0.05 were statistically significant. Variables that were found to be statistically significant (p-value < 0.2) in the bivariate analysis were included in the initial model. The backward stepwise elimination method was used, whereby variables that did not contribute significantly to the model were removed one by one. A multivariable regression model was established, and multicollinearity was assessed. The goodness-of-fit for the final model was assessed using the Pearson chi-square goodness-of-fit test. | Page 10 |
| **Results Presentation** | | |
| Please clearly distinguish:  Any-dose uptake: 53.3%  Complete series coverage: 20.9% among vaccinated women (90/229 ≈ 21%), which corresponds to approximately 21% (90/430) of the total sample.  Present these proportions consistently to avoid confusion. | Thank you, dear reviewer, for this comment. We have addressed this accordingly and refined this section, “**HBV Vaccination and Infection Status,”** as follows:  **Before adjustment**  *More than half, 53.3% (229/430) of the participants had received the HBV vaccine, and one in five, 20.9% (90/229) had received three doses (Table 3).*  **After corrections**  *More than half, 53.3% (229/430) of the participants had received the HBV vaccine, and about 39.3% (90/229) had received three doses (Table 3).* | Page 14 and 15 |
| **Contextual Comparison** |  |  |
| The comparison with uptake rates in Hong Kong and the United States is appropriate; however, please contextualize these findings by acknowledging differences in income level, national immunization policies, and routine birth-dose/adult vaccination programs to avoid over-interpretation. | Thank you, dear reviewer, for this comment. We have incorporated these suggestions into the discussion section.  **Before incorporating the comments**  *While the higher prevalence of vaccine uptake in Hong Kong and the USA was attributed to proper health-seeking behaviors, including antenatal visits of more than 8 times by pregnant mothers. During these visits, the mothers were routinely screened for HBV and encountered healthcare providers who advised them to get vaccinated. Additionally, the vaccination programs for pregnant mothers were free of charge, which could have contributed to a higher vaccination rate.*  **After incorporating the comments**  *While the higher prevalence of vaccine uptake in Hong Kong and the USA was attributed to the differences in income level, national immunization policies, and routine vaccination programs, and proper health-seeking behaviors, including antenatal visits of more than 8 times by pregnant mothers. During these visits, the mothers were routinely screened for HBV and encountered healthcare providers who advised them to get vaccinated. Additionally, the vaccination programs for pregnant mothers were free of charge, which could have contributed to a higher vaccination rate.* | Page 24 |
