# Supplementary material for: Hepatitis B vaccine uptake and associated factors among pregnant women attending antenatal care at Gulu Regional Referral Hospital, Northern Uganda
Source: PLoS One. 2026 Jun 2;21(6):e0350568. doi: 10.1371/journal.pone.0350568 (PMC13229301; doi:10.1371/journal.pone.0350568)
Supplement: Attachment — Submitted filename: Response_to_Reviewers_auresp_2.docx [file pone.0350568.s004.docx]

Makerere University,

School of Public Health

11.05.2026

**The Academic Editor**

**PLOS ONE**

Dear Editors,

We appreciate the opportunity to have our manuscript reviewed and for the valuable comments provided. We have fully addressed the comments as indicated below.

| **Comment** | **Response** | **Page No.** |
| --- | --- | --- |
| **Reviewer one** | | |
| 1. In the abstract, HBV is abbreviated as "HPV". Kindly correct | Thanks for the comments. This has been corrected in the abstract and changed from HPV to HBV as guided. | Page 2 |
| 2. The reference list has so many web pages of WHO/MoH, which could be minimised | Thank you for the comment. We acknowledge the concern regarding the number of web-based references from the WHO and the Ministry of Health. However, the cited references were obtained from different source documents and webpages, each providing specific information relevant to particular sections of the manuscript. Therefore, we are unable to remove them arbitrarily, as doing so may affect the accuracy, traceability, and proper attribution of the information presented. | Page 31,32, and 33 |
| 3. Use PLoS One referencing style (X), Vancouver | Thank you for this comment. The whole document is already referenced by the Vancouver style as instructed | ALL |
| 4. In the discussion, ANC didn’t need to be spelled out in full, just since already abbreviated earlier on | Thank you for this comment. This has been corrected and clearly abbreviated as instructed by our reviewer. | Page 24 |
